# Is Tuberculosis Treatment Really Free in China? A Study Comparing Two Areas with Different Management Models

**DOI:** 10.1371/journal.pone.0126770

**Published:** 2015-05-20

**Authors:** Sangsang Qiu, Hongqiu Pan, Simin Zhang, Xianzhen Peng, Xianzhi Zheng, Guisheng Xu, Min Wang, Jianming Wang, Hui Lu

**Affiliations:** 1 Department of Epidemiology and Biostatistics, School of Public Health, Nanjing Medical University, Nanjing, China; 2 Department of Tuberculosis, Third Hospital of Zhenjiang City, Zhenjiang, China; 3 Department of Social Medicine and Health Education, School of Public Health, Nanjing Medical University, Nanjing, China; 4 The Innovation Center for Social Risk Governance in Health, Nanjing, China; Johns Hopkins Bloomberg School of Public Health, UNITED STATES

## Abstract

**Objective:**

China has implemented a free-service policy for tuberculosis. However, patients still have to pay a substantial proportion of their annual income for treatment of this disease. This study describes the economic burden on patients with tuberculosis; identifies related factors by comparing two areas with different management models; and provides policy recommendation for tuberculosis control reform in China.

**Methods:**

There are three tuberculosis management models in China: the tuberculosis dispensary model, specialist model and integrated model. We selected Zhangjiagang (ZJG) and Taixing (TX) as the study sites, which correspond to areas implementing the integrated model and dispensary model, respectively. Patients diagnosed and treated for tuberculosis since January 2010 were recruited as study subjects. A total of 590 patients (316 patients from ZJG and 274 patients from TX) were interviewed with a response rate of 81%. The economic burden attributed to tuberculosis, including direct costs and indirect costs, was estimated and compared between the two study sites. The Mann-Whitney U Test was used to compare the cost differences between the two groups. Potential factors related to the total out-of-pocket costs were analyzed based on a step-by-step multivariate linear regression model after the logarithmic transformation of the costs.

**Results:**

The average (median, interquartile range) total cost was 18793.33 (9965, 3200-24400) CNY for patients in ZJG, which was significantly higher than for patients in TX (mean: 6598.33, median: 2263, interquartile range: 983–6688) (Z = 10.42, P < 0.001). After excluding expenses covered by health insurance, the average out-of-pocket costs were 14304.4 CNY in ZJG and 5639.2 CNY in TX. Based on the multivariable linear regression analysis, factors related to the total out-of-pocket costs were study site, age, number of clinical visits, residence, diagnosis delay, hospitalization, intake of liver protective drugs and use of the second-line drugs.

**Conclusion:**

Under the current “free of diagnosis and treatment” policy, the financial burden remains heavy on tuberculosis patients. Policy makers need to consider appropriate steps to lessen the burden of out-of-pocket costs for tuberculosis patients in China and how best to improve service delivery for poor patients.

## Introduction

Tuberculosis (TB) is a global health problem and remains a major cause of morbidity and mortality in developing countries [1]. China has the world's second largest tuberculosis epidemic, accounting for 12% of the total number of cases. In 2012, there were 1 million new cases and 44000 tuberculosis-related deaths in China [1]. The rising multidrug-resistant (MDR) tuberculosis is increasing an already heavy burden on China’s health system [2, 3].

Tuberculosis has been regarded as a “poverty-related disease” due to the association with poverty and malnutrition, which are more prevalent in developing countries. For example, in South Africa, tuberculosis is referred to as a “barometer of poverty” [4]. Tuberculosis-affected patients and their family members face many economic and social problems, such as high medical costs, loss of productivity, stigmatization and social isolation [5, 6]. In 1992, China initiated its modern National Tuberculosis Control Program (NTP) with directly observed treatment, short-course (DOTS) [7]. Recently, especially after the outbreak of Severe Acute Respiratory Syndrome in 2003, the Chinese government has taken a series of measures to strengthen its public health system with great efforts towards tuberculosis control. By 2005, China achieved the global targets for tuberculosis control with 100% DOTS coverage and over 90% treatment success [8]. Each year in China, more than 1 million tuberculosis patients receive DOTS therapy [9]. To reduce the financial barriers to and burdens on patients seeking essential healthcare, a “free-TB service policy” has been implemented gradually throughout the country [10, 11]. Under this policy, tuberculosis suspects are provided a free diagnosis and anti-tuberculosis treatment, including a free chest X-ray examination, sputum smear test and designated first-line anti-tuberculosis drugs [12]. Initially, the free-service policy was only performed for sputum smear-positive patients. Now it has expanded to sputum smear-negative patients. Moreover, the government has taken more measures to reduce the patient burden, including the establishment of universal health coverage and increasing the reimbursement rate for patients with tuberculosis [13]. The central government's spending on tuberculosis control increased from 40 million Chinese Yuan (CNY) in 2001 to 580 million CNY in 2010 [14]. China has established universal health coverage for 830 million rural residents through the rapid expansion of the New Cooperative Medical Scheme (NCMS). Moreover, a free-service policy has been gradually adopted in order to lighten the economic burden of patients with tuberculosis. Despite these policy changes, previous studies have revealed that patients still bear a high financial burden, which could affect their health care-seeking behaviors and treatment outcomes [10, 15–18]. The revenue-driven practices in some health facilities, such as over-prescription of medication, poor referral and high hospitalization rates not only influence a patient’s economic burden but also affects the entire tuberculosis control program [19, 20].

There are three common types of tuberculosis management models in China: the tuberculosis dispensary model, specialist model and integrated model [21]. For the dispensary model, patients are diagnosed and treated in tuberculosis dispensaries, which are usually affiliated with local CDC (Center for Disease Control and Prevention). The specialist model is similar to the dispensary model, but a specialized hospital is also responsible for treating the patients. For the integrated model, tuberculosis diagnosis and treatment is integrated into a general hospital which is referred to as a “designated hospital” [22].

This study describes the economic burden on patients with tuberculosis, identifies related factors by comparing two areas with different management models, and provides a policy recommendation for the tuberculosis control system in China.

## Subjects and Methods

### Ethical consideration

The Institutional Review Board (IRB) of Nanjing Medical University approved the study. Written informed consent was obtained from all of the participants. Ethical practices were used throughout the study period.

### Study design and settings

A descriptive study was performed in two counties of Jiangsu Province, China. One county, Zhangjiagang (ZJG), is located in the southern part of Jiangsu, which is a relatively rich area and ranked as the third most developed county of China in 2013. Another county, Taixing (TX), is located in the middle of Jiangsu, which is a moderately developed county. In 2010, the per capita GDP was 129535 Chinese Yuan (CNY) in ZJG and 36994 CNY in TX (http://www.jssb.gov.cn/). In the same year, the per capita GDP was 30567 CNY in China (http://www.stats.gov.cn/tjsj/ndsj/). ZJG adopted the integrated model, and the diagnosis and treatment of tuberculosis are provided by the designated county-level general hospital. TX implemented the dispensary model, and tuberculosis patients are diagnosed and treated at the local CDC (tuberculosis dispensary). In ZJG, the free service covered the first-line anti-tuberculosis drugs, four liver function tests, three chest X-ray examinations and all sputum smear microscopy tests. Moreover, each patient could receive a reimbursement of 150 CNY for liver protection drugs and each migrant patient with tuberculosis could receive an additional subsidy of 120 CNY. In TX, the free service policy covered the first-line anti-tuberculosis drugs, two liver function tests, one chest X-ray examination and all sputum smear microscopy tests. Hospitalization and second-line anti-tuberculosis drugs were not free of charge in either area. Baseline characteristics of the two study settings were listed in Table 1.

**Table 1:** Baseline characteristics of the two study settings in 2010.

Variables	ZJG	TX
Area (km^2^)	999	1172
Number of districts/towns	9	16
Registered residents at the end of 2010	905100	1196200
Population density (person/km^2^)	915	1023
Number of births	7763	9306
Number of deaths	6366	11308
Per capita net income of rural residents (CNY)	14658	9338
Number of health technical personnel	5898	3785
Tuberculosis management model	Integrated model	TB dispensary model
Specific staffs working for tuberculosis	7	8
Staffs with bachelor degree or above	5	3

### Study subjects

In ZJG, 23 villages were selected as study sites based on a random cluster sampling method. Three hundred and forty patients who were diagnosed with pulmonary tuberculosis since January 2010 were recruited for the investigation. The patients had already completed the standard anti-tuberculosis treatment prior to March 2013. In TX, we randomly selected 34 villages as study sites. Three hundred and ninety patients who were diagnosed with pulmonary tuberculosis since January 2010 were enrolled. Patients with MDR-TB were excluded from the study. Due to the gradually evolving tuberculosis management models in TX since 2012, the recruited patients were limited to those who had finished anti-tuberculosis treatment prior to 2012. A total of 590 patients (316 patients from ZJG and 274 patients from TX) were successfully interviewed and involved in the analysis with a response rate of 81%.

### Data collection

With the patients' informed consent, trained investigators interviewed them at their homes using a structured questionnaire to gather information, including demographic characteristics, socioeconomic status, health insurance, health care-seeking history and costs related to tuberculosis diagnosis and treatment.

### Estimation of economic burden

The economic burden of the patients with tuberculosis consisted of direct and indirect costs. The direct costs were medical costs related to tuberculosis diagnosis and treatment (including outpatient and inpatient expenses), transportation, accommodation and food [23, 24]. The indirect costs were calculated as a loss of income due to an inability to work due to the disease. We examined the loss of income (or decreased earning ability) for both patients of the labor force and their caregivers [23]. The total out-of-pocket costs were calculated by excluding reimbursement through medical insurance. The out-of-pocket direct costs were direct costs calculated by excluding reimbursement through medical insurance. The costs are expressed in the Chinese Yuan (CNY). In 2014, 1 CNY was equal to 0.16 USD. In order to calculate the costs for outpatient visits to the doctor, the number and costs of the visits made for tuberculosis diagnosis and treatment were obtained from the patients and their family members. If the patients could not remember the costs of the doctor’s visits, medical records and invoices were checked. The hospitalization cost of the patients was computed based on the cost that they paid when being discharged from the hospital. Non-medical, direct costs included transportation; commuting and food costs (of patients and their families) during their visit to health facilities; the cost of purchasing extra health products that were required due to the tuberculosis; the cost of residing in other cities for treatment and nursing patients at home. Indirect costs depended on daily income; the number of sick leaves; the average daily income of each patient's companion; and the duration of absence from work resulting from nursing and caring for the patient. The individuals’ wages were used to calculate lost income. For patients who were not willing to declare their income and also for housekeepers, the local average daily wage was used.

### Data analysis

We used EpiData 3.1 (http://epidata.dk) software to input data with double entry for consistency. Statistical analyses was performed using STATA 12.0 software (College Station, Texas, USA). Considering the distribution of costs, we used the mean and median (interquartile range, IQR) to describe a patient’s economic burden. The Mann-Whitney U Test was used to compare the cost difference between the two groups. The cost was logarithmically transformed as the dependent variable, and a multivariate linear regression model was performed to analyze potential factors related to the out-of-pocket costs of patients with tuberculosis. We included product terms in our model to individually account for each possible two-way interaction, considering as statistically significant those interactions with a P-value < 0.05.

## Results

### General information

This study recruited 590 tuberculosis patients, including 425 men (72.0%) and 165 women (28.0%). Among them, 316 (53.6%) resided in ZJG, and 274 (46.4%) resided in TX. In ZJG, there were many young, migrant patients with tuberculosis, and fewer patients had a treatment history. The proportion of hospitalized patients and patients taking liver protective drugs were also higher in ZJG than TX (Table 2).

**Table 2:** Patient characteristics.

Variables	ZJG	TX	χ^2^	*P*
	N (%)	N (%)		
**Sex**				
Male	191(69.71)	234(74.05)	1.37	0.241
Female	83(30.29)	82(25.95)		
**Age (year)**				
<60	211(77.01)	166(52.53)	38.11	**<0.001**
≥60	63(22.99)	150(47.47)		
**Residence**				
Local patient	149(54.38)	310(98.10)	162.41	**<0.001**
Migrant patient	125(45.62)	6(1.90)		
**Education**				
Illiterate	24(8.76)	65(20.57)	42.79	**<0.001**
Primary school	70(25.55)	90(28.48)		
Junior middle school	106(38.69)	129(40.82)		
Senior high school	44(16.06)	27(8.54)		
University or above	30(10.95)	5(1.58)		
Treatment history*				
Yes	21(7.95)	42(14.29)	5.57	**0.018**
No	243(92.05)	252(85.71)		
**Hospitalization**				
Yes	100(36.50)	49(15.51)	34.25	**<0.001**
No	174(63.50)	267(84.49)		
**Adverse drug reactions**				
Yes	74(27.99)	123(38.92)	9.37	**0.002**
No	200(72.99)	193(61.08)		
**Intake of liver protective drugs**				
Yes	168(61.31)	61(19.30)	109.06	**<0.001**
No	106(38.69)	255(80.70)		

*: some missing data.

### Patient economic burden

As shown in Table 3, the average (median, IQR) total costs was 18793.33 (9965, 3200–24400) CNY for patients in ZJG, which was significantly higher than for patients in TX (mean: 6598.33, median: 2263, IQR: 983–6688) (Z = 10.42, P < 0.001). After excluding the expenses covered by health insurance, the total out-of-pocket costs was 14304.4 CNY in ZJG and 5639.2 CNY in TX. The median (IQR) ratio of total out-of-pocket cost to the annual family income was 20.5% (7.5%-58.7%) in ZJG and 10.3% (3.9%-31.2%) in TX (Table 3).

**Table 3:** Tuberculosis related economic burden of patients in ZJG and TX.

Category	ZJG (n = 274)		TX (n = 316)		Z^#^	*P*
	Mean	Median(IQR)*	Mean	Median(IQR)*		
**Total costs**	18793.3	9965(3200–24400)	6598.3	2263(983–6688)	10.42	**<0.001**
Total out-of pocket costs	14304.4	7130(2500–19100)	5639.2	2183(894–5554)	8.87	**<0.001**
**Direct costs**	11936.9	4590(2024–14600)	3983.1	1200(520–2845)	11.55	**<0.001**
Outpatient	3484.8	2000(1000–4060)	1379.6	800(381–1500)	8.42	**<0.001**
Hospitalization	6978.6	0(0–8045)	2145.3	0(0–0)	6.13	**<0.001**
Transportation	636.4	200(100–650)	293.1	200(100–400)	1.79	0.074
Accommodation and food	837.1	100(0–800)	165.2	0(0–0)	10.60	**<0.001**
Out-of-pocket direct costs	7448.0	3315(1200–8570)	3024.0	1086(480–2456)	9.768	**<0.001**
Outpatient	2551.0	1100(500–3000)	1219.0	700(300–1245)	5.45	**<0.001**
Hospitalization	3423.4	0(0–2800)	1346.7	0(0–0)	5.97	**<0.001**
Transportation	636.4	200(100–650)	293.1	200(100–400)	1.79	0.074
Accommodation and food	837.1	100(0–800)	165.2	0(0–0)	10.60	**<0.001**
**Indirect costs**	6856.4	1575(0–9000)	2615.2	500(250–2025)	1.14	0.256
Patients	6067.3	300(0–8000)	2151.0	250(150–1500)	-2.20	**0.028**
Accompanied family members	789.1	0(0–500)	464.2	150(0–250)	-3.73	**<0.001**

*IQR: Interquartile range;

^#^Mann-Whitney rank test.

In ZJG, the average direct costs was 11936.9 CNY with a median (IQR) of 4590 (2024–14600). In TX, the average direct costs was 3983.1 CNY with a median (IQR) of 1200 (520–2845). When we excluded the expenses covered by health insurance, the average out-of-pocket direct costs was 7448.0 CNY [median (IQR): 3315 (1200–8570)] in ZJG and 3024.0 CNY [median (IQR): 1086 (480–2456)] in TX (Table 3).

The mean indirect costs was 6856.4 CNY [median (IQR):1575 (0–9000)] in ZJG, including the productivity loss by patients (mean: 6067.3, median: 300, IQR: 0–800) and accompanied family members (mean: 789.1, median: 0, IQR: 0–500). In TX, the indirect costs averaged 2615.2 CNY [median (IQR): 500 (250–2025)], including the productivity loss by patients (mean: 2151, median: 250, IQR:150–1500) and accompanied family members (mean: 464.2, median: 150, IQR:0–250) (Table 3).

### Factors contributing to out-of-pocket costs

The common factors contributing to the total out-of-pocket costs in these two areas were diagnosis delay, hospitalization, and intake of liver protective drugs and second-line drugs. A univariate analysis also identified several peculiar features related to the total out-of-pocket costs in TX, including the patients’ age, treatment history, adverse drug reactions and the number of clinical visits (Table 4).

**Table 4:** Factors related to the total out-of-pocket costs of patients in ZJG and TX.

Variables	ZGJ				TX			
	N	Median(IQR)*	Z^#^	*P*	N	Median(IQR)*	Z^#^	*P*
**Sex**								
Male	191	6700(2300–19900)	-1.090	0.276	234	2365(900–6200)	1.357	0.175
Female	83	8080(3200–18590)			82	1578(860–4800)		
**Age**								
<60	211	7200(3000–21700)	1.735	0.083	166	2800(1180–6475)	3.178	**0.002**
≥60	63	6800(1950–13540)			150	1600(790–4300)		
**Residence**								
Local patient	149	8500(2000–20396)	0.049	0.961	310	2133(888–5700)	0.316	0.752
Migrant patient	125	6518(3030–16400)			6	2280(2050–2750)		
**Sputum smear test**								
Positive	102	5999(2900–15350)	0.972	0.331	182	2474(1045–6150)	1.803	0.071
Negative	172	8650(2340–20100)			134	1663(730–5400)		
**treatment history** ^^^								
Yes	21	7060(3900–23200)	-0.678	0.498	42	1513(623–3260)	2.588	**0.010**
No	243	7050(2400–19100)			252	2365(1042–6208)		
**Adverse drug reactions**								
Yes	74	8150(3000–23000)	1.274	0.203	123	3150(1207–7550)	3.020	**0.003**
No	200	6860(2390–17265)			193	1610(820–4840)		
**Abnormal liver function**								
Yes	40	6860(2025–23100)	0.263	0.792	87	2600(1127–7550)	1.864	0.062
No	234	7130(2500–18590)			229	1850(830–5400)		
**Clinical visits** ^^^								
<7 times	157	6920(2500–17250)	0.546	0.585	121	1050(575–3213)	6.112	**<0.001**
≥7 times	109	7200(2380–19364)			194	2920(1486–6300)		
**Diagnosis delay** ^^^								
<2weeks	172	6110(2045–15425)	2.970	**0.003**	241	1680(770–4630)	4.115	**<0.001**
≥2weeks	98	11065(3700–23200)			75	4320(1600–8900)		
**Hospitalization**								
Yes	100	15500(8350–29250)	8.119	**<0.001**	49	10272(5588–24520)	8.816	**<0.001**
No	174	3800(1298–10600)			267	1600(780–3570)		
**Intake of liver protective drugs**								
Yes	168	8150(3027–22500)	2.645	**0.008**	61	3340(1207–10800)	2.725	**0.006**
No	106	5900(1820–14650)			255	2000(840–5200)		
**Intake of second-line drugs**								
Yes	58	10325(3700–26680)	2.029	**0.0423**	156	3188(1416–7632)	4.990	**<0.001**
No	216	6785(2300–16050)			160	1341(651–4413)		

*IQR: interquartile range;

^#^Mann-Whitney rank test;

^^^some missing data.

We further performed a step-by-step linear regression analysis by including multiple variables in the model. Significant factors related to the total out-of-pocket costs were study setting (t = -3.10, P = 0.002), age (t = -4.04, P < 0.001), number of clinical visits (t = 4.46, P < 0.001), residence (t = 3.19, P = 0.002), diagnosis delay (t = 3.47, P = 0.001), hospitalization (t = 15.04, P < 0.001), intake of liver protective drugs (t = 2.78, P = 0.006) and intake of second-line drugs (t = 2.87, P = 0.004) (Table 5).

**Table 5:** Multivariate linear regression analysis on factors related to the total out-of-pocket costs*.

Variables	Coefficient	95% CI	*t*	*P*
**Study setting**				
ZJG	Ref.			
TX	-0.382	-0.624, -0.139	-3.10	**0.002**
**Age group**				
<60	Ref.			
≥60	-0.403	-0.598, -0.207	-4.04	**<0.001**
**Clinical visits**				
<7 times	Ref.			
≥7 times	0.404	0.226, 0.582	4.46	**<0.001**
**Residence**				
Local patient	Ref.			
Migrant patient	0.433	0.166, 0.700	3.19	**0.002**
**Diagnosis delay**				
<2 weeks	Ref.			
≥2 weeks	0.341	0.148, 0.534	3.47	**0.001**
**Hospitalization**				
No	Ref.			
Yes	1.672	1.454, 1.891	15.04	**<0.001**
**Intake of second-line drugs**				
No	Ref.			
Yes	0.271	0.079, 0.462	2.78	**0.006**
**Intake of liver protective drugs**				
No	Ref.			
Yes	0.289	0.091, 0.488	2.87	**0.004**

*Total out-of-pocket costs was logarithmically transformed

A significant interaction was found between the number of clinical visits and study setting (P_interaction_ = 0.001). We then performed a multivariate linear regression analysis stratified by study setting. As shown in Table 6, age, residence, diagnosis delay and hospitalization entered into the model in ZJG. The number of clinical visits, age, diagnosis delay, hospitalization, intake of liver protective drugs and intake of second-line drugs entered into the model in TX.

**Table 6:** Multivariate linear regression analysis on factors related to the total out-of-pocket costs stratified by study setting*.

Variables	Coefficient	95% CI	*t*	*P*
**ZJG**				
**Age group**				
<60	Ref.			
≥60	-0.607	-0.978, -0.235	-3.22	**0.001**
**Residence**				
Local patient	Ref.			
Migrant patient	0.486	0.160, 0.812	2.93	**0.004**
**Diagnosis delay**				
<2 weeks	Ref.			
≥2 weeks	0.326	0.038, 0.614	2.23	**0.026**
**Hospitalization**				
No	Ref.			
Yes	1.724	1.411, 2.038	10.83	**<0.001**
**TX**				
**Age group**				
<60	Ref.			
≥60	-0.345	-0.561, -0.129	-3.15	**0.002**
**Clinical visits**				
<7 times	Ref.			
≥7 times	0.579	0.353, 0.805	5.05	**<0.001**
**Diagnosis delay**				
<2 weeks	Ref.			
≥2 weeks	0.419	0.161, 0.677	3.20	**0.002**
**Hospitalization**				
No	Ref.			
Yes	1.664	1.365, 1.962	10.97	**<0.001**
**Intake of second-line drugs**				
No	Ref.			
Yes	0.397	0.178, 0.616	3.57	**<0.001**
**Intake of liver protective drugs**				
No	Ref.			
Yes	0.386	0.112, 0.659	2.77	**0.006**

*Total out-of-pocket costs was logarithmically transformed.

## Discussion

Tuberculosis has historically been endemic in China, primarily due to limited health resources and government neglect [14]. In recent years, the political commitment to public health has significantly increased [25]. China is moving towards primary health care based on community services [14]. For example, the government pays sustainable funds per person for the prevention of disease, and some funds are allotted to tuberculosis control. Financial concern is the most important factor guiding health care seeking behavior among the Chinese population [26–28]. However, as demonstrated in the current study and previous reports [10, 13, 16, 20], most of the patients have complained about paying a major part of the treatment cost through out of pocket payments. To modify the current tuberculosis control strategies, policy makers must identify the factors related to patient economic burden.

In this study, we selected two counties from the Jiangsu province using different tuberculosis management models. ZJG adopted the integrated model. One advantage of this model is that it can shorten the delay time of diagnosis and treatment [22]. Previous studies have demonstrated that multiple clinical visits and a delay of diagnosis can increase the financial burden on patients and influence the successful control of tuberculosis [29]. Patient delay also occurs due to a lack of knowledge or negligence in seeking health care services until their symptoms deteriorate [30–32]. The inability of doctors to diagnose tuberculosis during the patient’s early visits leads to an institutional delay [15]. Suspected patients are usually provided symptomatic treatment resulting in increased expenses.

The WHO recommends outpatient treatment for non-complicated tuberculosis patients. However, unnecessary hospitalization has been reported, which not only leads to higher inpatient expense, but also results in extra costs related to accommodation and transportation. The integrated tuberculosis management model may increase the hospitalization rate of suspected patients in the designated hospital. A study demonstrated that in tuberculosis patients without concomitant disorders, the initiation of treatment at a hospital adversely influenced the outcome of treatment, as reflected by the percentage of completers [33]. However, for patients with complicated or severe symptoms, hospitalization can be advantageous.

The intake of liver protective drugs and second-line anti-tuberculosis drugs were two common factors influencing a patient’s economic burden. One of the most adverse reactions caused by anti-tuberculosis drugs is drug-induced liver damage. To avoid this reaction, clinical doctors in China usually prescribe protective drugs. These drugs include herbals, manufactured herbal products, and combinations of vitamins and other non-herbal substances (although their preventive effects have not been proven) [34, 35]. Liver-protecting drugs are not free. Over-prescription of these medications also increases the patients’ out-of-pocket costs [10]. Currently, in many settings in China, the prescription of second-line drugs primarily depends on the treatment history of the patients rather than the drug susceptibility test. As reported in one cross-sectional study conducted in five provinces within China, approximately 23.8% of the patients had a history of taking second-line drugs [36]. Even worse, there was no significant association between the prescription of second-line drugs and the drug resistant statuses of the patients. Abuse of second-line drugs merely based on clinical judgments not only increases the risk of MDR-TB and XDR-TB but also results in extra economic burden on the patients [17].

In addition to the medical costs due to the disease, the loss of working time experienced by patients and their family members should not be ignored. A Dutch study demonstrated that patients lose (on average) 2.7 months (81 days) of productive days due to tuberculosis [37]. In developed areas, the indirect costs would be much higher. For example, in our study, patients in ZJG had nearly four times higher indirect costs compared with patients in TX, which might be attributed to a higher level of economic development and per capita income in ZJG.

Besides the common factors related to the total out-of-pocket costs in ZJG and TX, the univariate analysis revealed that age, treatment history, adverse drug reactions and number of clinical visits were significant factors in TX but not in ZGJ. These particular factors may play different roles in a patient’s economic burden in different areas. For example, a significant interaction was found between the clinical visits and study setting. The number of clinical visits was more important in TX as compared with ZJG. Also, the relatively small sample size in ZJG may reduce the statistical power, resulting in nonsignificant findings.

Some limitations existed in this study. First, we selected ZJG and TX as the study sites representing areas using the integrated model and TB dispensary model. However, the socio-economic status and local health system may also influence a patient’s direct and indirect costs. The cost differences between these two areas could not be completely attributed to the disparity of tuberculosis management models. Second, although we estimated the expenses by interviewing patients and their family members and checking medical records, recall bias should not be neglected. Third, the mean costs may not be reflective of the median. Considering the non-normal distribution of the costs, we used the mean and median (interquartile range, 25%-75%) to describe a patient’s economic burden. We also applied a non-parametric test to compare the cost differences between groups. A logarithmic transformation was performed to normalize the distribution when we performed a step-by-step multivariate linear regression analysis.

In this study, we revealed several common and specific factors affecting the economic burden of patients’ with tuberculosis. Health policy makers should consider these factors by enhancing financial support, strengthening health facilities and involving human resources to achieve success in tuberculosis control. Tuberculosis control in China is a long-term, public health challenge and needs the support of affordable and sustainable health resources. Community health resources within a strengthened health system might be the best approach [38]. Evidence-based measures to improve healthcare-seeking behavior, reduce patient and detection delays, address financial and system barriers, improve the quality of direct observed therapy and increase the access to health promotion are urgently needed [39]. Policy makers need to further document their challenges when implementing tuberculosis management models [21].

## Conclusion

Under the current “free diagnosis and treatment” policy, the financial burden remains heavy on tuberculosis patients. Policy makers need to consider appropriate steps to lessen the burden of out-of-pocket costs for tuberculosis patients in China and how best to improve service delivery for poor patients.

## References

[1] WHO. Global tuberculosis report 2013: World Health Organization; 2013 Available: http://www.who.int/tb/publications/global_report/en/. Accessed 01 December 2014.

[2] Falzon D, Mirzayev F, Wares F, Baena IG, Zignol M, Linh N, et al Multidrug-resistant tuberculosis around the world: what progress has been made? Eur Respir J. 2015;45:150–60. 10.1183/09031936.00101814 . 25261327 PMC4318660

[3] Shao Y, Yang D, Xu W, Lu W, Song H, Dai Y, et al Epidemiology of anti-tuberculosis drug resistance in a Chinese population: current situation and challenges ahead. BMC Public Health. 2011;11:110 10.1186/1471-2458-11-110 . 21324205 PMC3045946

[4] Lutge E, Lewin S, Volmink J, Friedman I, Lombard C. Economic support to improve tuberculosis treatment outcomes in South Africa: a pragmatic cluster-randomized controlled trial. Trials. 2013;14:154 10.1186/1745-6215-14-154 . 23714270 PMC3680200

[5] Gurung GN, Chhetri PS, Jha N. Economic impact of pulmonary tuberculosis on patients and their families of Dharan municipality, Nepal. Nepal Med Coll J. 2012;14:196–8. . 24047014

[6] Liefooghe R, Michiels N, Habib S, Moran MB, De Muynck A. Perception and social consequences of tuberculosis: a focus group study of tuberculosis patients in Sialkot, Pakistan. Soc Sci Med. 1995;41:1685–92. . 8746868 10.1016/0277-9536(95)00129-u

[7] Xu B, Dong HJ, Zhao Q, Bogg L. DOTS in China—removing barriers or moving barriers? Health Policy Plan. 2006;21:365–72. 10.1093/heapol/czl019 . 16940302

[8] Wang L, Liu J, Chin DP. Progress in tuberculosis control and the evolving public-health system in China. Lancet. 2007;369:691–6. 10.1016/S0140-6736(07)60316-X . 17321314 PMC7134616

[9] Lv X, Tang S, Xia Y, Wang X, Yuan Y, Hu D, et al Adverse reactions due to directly observed treatment strategy therapy in chinese tuberculosis patients: a prospective study. PLOS One. 2013;8:e65037 10.1371/journal.pone.0065037 . 23750225 PMC3672195

[10] Pan HQ, Bele S, Feng Y, Qiu SS, Lu JQ, Tang SW, et al Analysis of the economic burden of diagnosis and treatment of tuberculosis patients in rural China. Int J Tuberc Lung Dis. 2013;17:1575–80. 10.5588/ijtld.13.0144 . 24200271

[11] Wei X, Chen J, Chen P, Newell JN, Li H, Sun C, et al Barriers to TB care for rural-to-urban migrant TB patients in Shanghai: a qualitative study. Trop Med Int Health. 2009;14:754–60. 10.1111/j.1365-3156.2009.02286.x . 19392747

[12] Liu Q, Smith H, Wang Y, Tang S, Wang Q, Garner P. Tuberculosis patient expenditure on drugs and tests in subsidised, public services in China: a descriptive study. Trop Med Int Health. 2010;15:26–32. 10.1111/j.1365-3156.2009.02427.x . 19917035

[13] Wei X, Zou G, Yin J, Walley J, Zhang X, Li R, et al Effective reimbursement rates of the rural health insurance among uncomplicated tuberculosis patients in China. Trop Med Int Health. 2015;20:304–11. 10.1111/tmi.12438 . 25430477

[14] Jia Z, Cheng S, Wang L. Tuberculosis control in China: striving for sustainability. Lancet. 2012;379:2149 10.1016/S0140-6736(12)60942-8 . 22682462

[15] Liu X, Thomson R, Gong Y, Zhao F, Squire SB, Tolhurst R, et al How affordable are tuberculosis diagnosis and treatment in rural China? An analysis from community and tuberculosis patient perspectives. Trop Med Int Health. 2007;12:1464–71. 10.1111/j.1365-3156.2007.01953.x . 18076553

[16] Xu B, Fochsen G, Xiu Y, Thorson A, Kemp JR, Jiang QW. Perceptions and experiences of health care seeking and access to TB care—a qualitative study in rural Jiangsu Province, China. Health Policy. 2004;69:139–49. 10.1016/j.healthpol.2003.11.006 . 15212861

[17] Xu W, Lu W, Zhou Y, Zhu L, Shen H, Wang J. Adherence to anti-tuberculosis treatment among pulmonary tuberculosis patients: a qualitative and quantitative study. BMC Health Serv Res. 2009;9:169 10.1186/1472-6963-9-169 . 19765290 PMC2753329

[18] Small P. Why India should become a global leader in high-quality, affordable TB diagnostics. Indian J Med Res. 2012;135:685–9. . 22771602 PMC3401703

[19] Zhan S, Wang L, Yin A, Blas E. Revenue-driven in TB control—three cases in China. Int J Health Plann Manage. 2004;19 Suppl 1:S63–78. 10.1002/hpm.778 . 15686061

[20] Long Q, Smith H, Zhang T, Tang S, Garner P. Patient medical costs for tuberculosis treatment and impact on adherence in China: a systematic review. BMC Public Health. 2011;11:393 10.1186/1471-2458-11-393 . 21615930 PMC3125370

[21] Wei X, Zou G, Walley J, Yin J, Lonnroth K, Uplekar M, et al China tuberculosis policy at crucial crossroads: comparing the practice of different hospital and tuberculosis control collaboration models using survey data. PLOS One. 2014;9:e90596 10.1371/journal.pone.0090596 . 24621996 PMC3951218

[22] Wei X, Zou G, Yin J, Walley J, Sun Q. Comparing patient care seeking pathways in three models of hospital and TB programme collaboration in China. BMC Infect Dis. 2013;13:93 10.1186/1471-2334-13-93 . 23425261 PMC3598790

[23] Barter DM, Agboola SO, Murray MB, Barnighausen T. Tuberculosis and poverty: the contribution of patient costs in sub-Saharan Africa—a systematic review. BMC Public Health. 2012;12:980 10.1186/1471-2458-12-980 . 23150901 PMC3570447

[24] Laokri S, Drabo MK, Weil O, Kafando B, Dembele SM, Dujardin B. Patients are paying too much for tuberculosis: a direct cost-burden evaluation in Burkina Faso. PLOS One. 2013;8:e56752 10.1371/journal.pone.0056752 . 23451079 PMC3581516

[25] Yip WC, Hsiao WC, Chen W, Hu S, Ma J, Maynard A. Early appraisal of China's huge and complex health-care reforms. Lancet. 2012;379:833–42. 10.1016/S0140-6736(11)61880-1 . 22386036

[26] Zhang T, Liu X, Bromley H, Tang S. Perceptions of tuberculosis and health seeking behaviour in rural Inner Mongolia, China. Health Policy. 2007;81:155–65. doi: S0168-8510(05)00302-7 [pii] 10.1016/j.healthpol.2005.12.009 . 16806562

[27] Liu Y, Hsiao WC, Eggleston K. Equity in health and health care: the Chinese experience. Soc Sci Med. 1999;49:1349–56. doi: S0277953699002075 [pii]. . 10509825 10.1016/s0277-9536(99)00207-5

[28] Zhang T, Tang S, Jun G, Whitehead M. Persistent problems of access to appropriate, affordable TB services in rural China: experiences of different socio-economic groups. BMC Public Health. 2007;7:19. doi: 1471-2458-7-19 [pii] 10.1186/1471-2458-7-19 . 17288593 PMC1805429

[29] Tobe RG, Xu L, Zhou C, Yuan Q, Geng H, Wang X. Factors affecting patient delay of diagnosis and completion of Direct Observation Therapy, Short-course (DOTS) among the migrant population in Shandong, China. Biosci Trends. 2013;7:122–8. . 23836035

[30] San Pedro A, Oliveira RM. [Tuberculosis and socioeconomic indicators: systematic review of the literature]. Rev Panam Salud Publica. 2013;33:294–301. . 23698179 10.1590/s1020-49892013000400009

[31] Sreeramareddy CT, Qin ZZ, Satyanarayana S, Subbaraman R, Pai M. Delays in diagnosis and treatment of pulmonary tuberculosis in India: a systematic review. Int J Tuberc Lung Dis. 2014;18:255–66. 10.5588/ijtld.13.0585 . 24670558 PMC4070850

[32] Li Y, Ehiri J, Tang S, Li D, Bian Y, Lin H, et al Factors associated with patient, and diagnostic delays in Chinese TB patients: a systematic review and meta-analysis. BMC Med. 2013;11:156 10.1186/1741-7015-11-156 . 23819847 PMC3699418

[33] Karasulu AL, Altin S, Dalar L, Sokucu SN, Ozkan P. Can hospitalization provide better compliance in smear positive tuberculosis patients? Tuberk Toraks. 2009;57:277–81. . 19787466

[34] Ho MJ. Perspectives on tuberculosis among traditional Chinese medical practitioners in New York City's Chinatown. Cult Med Psychiatry. 2006;30:105–22. 10.1007/s11013-006-9010-6 . 16721672

[35] Liu Q, Garner P, Wang Y, Huang B, Smith H. Drugs and herbs given to prevent hepatotoxicity of tuberculosis therapy: systematic review of ingredients and evaluation studies. BMC Public Health. 2008;8:365 10.1186/1471-2458-8-365 . 18939987 PMC2576232

[36] Xu B, Zhao Q, Hu Y, Shi Y, Wang W, Diwan VK. Experiences in anti-tuberculosis treatment in patients with multiple previous treatments and its impact on drug resistant tuberculosis epidemics. Glob Health Action. 2014;7:24593 10.3402/gha.v7.24593 . 25138531 PMC4138495

[37] Kik SV, Olthof SP, de Vries JT, Menzies D, Kincler N, van Loenhout-Rooyakkers J, et al Direct and indirect costs of tuberculosis among immigrant patients in the Netherlands. BMC Public Health. 2009;9:283 10.1186/1471-2458-9-283 . 19656370 PMC2734849

[38] Raviglione M, Zumla A, Marais B, Horton R, Motsoaledi A. A sustainable agenda for tuberculosis control and research. Lancet. 2012;379:1077–8. 10.1016/S0140-6736(12)60373-0 . 22444391

[39] Li Y, Ehiri J, Oren E, Hu D, Luo X, Liu Y, et al Are we doing enough to stem the tide of acquired MDR-TB in countries with high TB burden? Results of a mixed method study in Chongqing, China. PLOS One. 2014;9:e88330 10.1371/journal.pone.0088330 . 24505476 PMC3914979

